# Disease correlation network: a computational package for identifying temporal correlations between disease states from Large-Scale longitudinal medical records

**DOI:** 10.1093/jamiaopen/ooz031

**Published:** 2019-08-23

**Authors:** Huaiying Lin, Ruichen Rong, Xiang Gao, Kashi Revanna, Michael Zhao, Petar Bajic, David Jin, Chengjun Hu, Qunfeng Dong

**Affiliations:** 1 Center for Biomedical Informatics, Stritch School of Medicine, Loyola University Chicago, Maywood, Illinois, USA; 2 Department of Public Health Sciences, Stritch School of Medicine, Loyola University Chicago, Maywood, Illinois, USA; 3 Department of Urology, Stritch School of Medicine, Loyola University Chicago, Maywood, Illinois, USA; 4 Department of Electrical Engineering & Computer Sciences, Northwestern University, Evanston, Illinois, USA; 5 Depatment of Anatomy and Embryology, Wuhan University School of Medicine, Wuhan, Hubei, China

**Keywords:** electronic medical record, survival analysis, temporal correlation

## Abstract

**Objective:**

To provide an open-source software package for determining temporal correlations between disease states using longitudinal electronic medical records (EMR).

**Materials and Methods:**

We have developed an *R*-based package, Disease Correlation Network (DCN), which builds retrospective matched cohorts from longitudinal medical records to assess for significant temporal correlations between diseases using two independent methodologies: Cox proportional hazards regression and random forest survival analysis. This optimizable package has the potential to control for relevant confounding factors such as age, gender, and other demographic and medical characteristics. Output is presented as a DCN which may be analyzed using a JavaScript-based interactive visualization tool for users to explore statistically significant correlations between disease states of interest using graph-theory-based network topology.

**Results:**

We have applied this package to a longitudinal dataset at Loyola University Chicago Medical Center with 654 084 distinct initial diagnoses of 51 conditions in 175 539 patients. Over 90% of disease correlations identified are supported by literature review. DCN is available for download at https://github.com/qunfengdong/DCN.

**Conclusions:**

DCN allows screening of EMR data to identify potential relationships between chronic disease states. This data may then be used to formulate novel research hypotheses for further characterization of these relationships.

## OBJECTIVES

The availability of large-scale electronic medical records (EMR) presents unprecedented opportunities to uncover previously unrecognized temporal correlations between disease states. We provide an open-source software package for investigators to identify these correlations using longitudinal EMR data. Such correlations may be used to generate novel research hypotheses for future prospective studies.

## BACKGROUND

Many temporal correlations between specific disease states are well recognized. For example, hypertension may increase the risk of heart disease.^1^ Traditionally, such temporal correlations have been identified through epidemiological studies focusing on specific pairs of diseases. With the availability of large-scale EMRs, there is unprecedented opportunity to uncover temporal disease correlations which have not previously been recogniz.

A computational method was recently applied to study temporal disease trajectories among chronic diseases using the Danish National Patient Registry.^2^ The algorithmic foundation of this method (the Danish method) is based on a key computational approach for identifying temporal correlations among diseases. Briefly, the Danish method utilizes the following principle: For every disease pair of *X* and *Y*, the exposed group is defined as all patients with diagnosis of disease *X*. For each patient in the exposed group, a set of gender, ethnicity and age-matched control subjects without disease *X* is selected to form the corresponding comparison group. The number of case subjects, with diagnosis of disease *Y*, is counted in both the exposed and the comparison groups. Then, by applying the binomial statistical test, the Danish method examines whether the proportion of case subjects is significantly higher in the exposed group than in the comparison group. If higher, the Danish method determines that the earlier diagnosis of disease *X* may have a significant temporal correlation with the later diagnosis of disease *Y*. The results of this approach provide the foundation for subsequent analysis of connecting significantly correlated individual disease pairs into trajectories consisting of multiple diseases.

Although the Danish method is a significant step toward uncovering unknown disease correlations, there are several inherent limitations to the approach. First, the amount of lag time between the onsets of different diseases is not characterized. For example, if a patient in the exposed group with disease *X* developed disease *Y* after 1 year, and another patient in the comparison group without disease *X* developed disease *Y* after 4 years, the Danish method would treat the two cases identically. This limitation occurs as a result of the Danish method’s categorical designation of the presence of disease *Y* within a 5-year window, rather than as a continuous time-to-event variable. Second, confounding factors cannot be easily accounted for by the Danish method as a result of simple stratifications. To account for the effects of gender and ethnicity, the Danish method requires identical gender and ethnicity between patients in the exposed and comparison groups. Such stratification may be plausible for categorical data (eg, gender and ethnicity), but is not suitable for continuous variables (eg, age, blood pressure, body mass index, etc.) which often cannot be stratified into workable categories. Finally, no readily available software package of the Danish method has been provided for use by other investigators.

Our approach aims to overcome the limitations of the Danish method by integrating two independent complementary approaches—Cox Proportional Hazard (Cox-PH) regression and Random Forest (RF) survival analysis. These approaches treat time to onset of disease as a continuous variable, and flexibly incorporate categorical and continuous covariates into the modeling of temporal relationships between disease pairs. The output of disease correlations may be further analyzed using our customized visualization tool. Our software package, Disease Correlation Network (DCN), is available for download at https://github.com/qunfengdong/DCN.

## SIGNIFICANCE

In this study, we developed an easy-to-use software which overcomes limitations of the Danish method and allows for the utilization of EMR data to identify previously unstudied correlations between chronic disease states. Our software package facilitates the formulation of novel research hypotheses to further characterize these relationships in a prospective manner.

## MATERIALS AND METHODS

The major components of this software package are described in the following sections: (i) extracting retrospective matched cohorts from EMRs, (ii) performing Cox-PH regression, (iii) performing RF survival analysis, and (iv) exploring the correlations between diseases of interest based on statistical significance and network topology using a customized interactive visualization tool.

### Extraction of retrospective matched cohorts from longitudinal medical records

**Figure 1.**
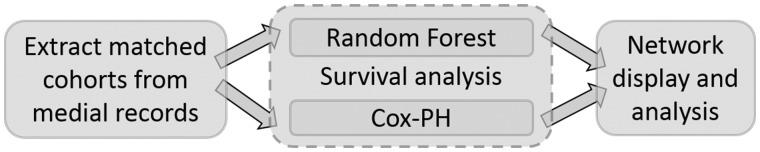
Flowchart of DCN (from left to right). The software constructs all possible disease pair cohorts from medical records, performs the proposed two methods of survival analysis and displays the Cox-PH regression results in an interactive network.

DCN requires an input file with at least six variables: patient de-identifier, disease code or name, the date the disease was diagnosed for the first time, patient age, gender, and race/ethnicity. Additional variables may be appended as confounding factors. The input data may be prepared from longitudinal EMRs. For every combination of disease pairs, DCN automatically builds retrospective cohorts for analysis by extracting exposed and comparison groups from the input data. For example, in order to examine whether the earlier diagnosis of disease *X* is correlated with the later diagnosis of disease *Y* (referred to as disease pair *X* → *Y*), subjects are designated in the exposed group as diagnosed with disease *X* (ie, subjects in the exposed group were exposed to disease *X*). For each subject in the exposed group, DCN randomly selects a matched subject in the comparison group in which the subjects were not exposed to disease *X*. The matching criteria in DCN are the same as in the Danish method—namely, that the subject in the exposed group and the matched subject in the comparison group have the same gender and ethnicity, and the diagnosis time of a random non-*X* disease is within 1 week of the diagnosis of *X* in the exposed group. A detailed workflow is shown in Figure 1.

Users may decide on several command-line parameters including difference in diagnosis time (the default is within 7 days), age difference between the exposed subject and the matched comparison subject (default is within 5 years), the maximum number of subjects in the exposed and comparison group (default is 10 000). More command-line options may be found on the GitHub page.

### Survival analysis using Cox-PH regression

Survival analysis, widely used in epidemiology, public health, and medicine, provides a natural framework for studying time-to-event data. A time-to-event variable is a measurement of time until a subject has an event of interest, such as heart attack, death, or cancer remission. The goal of survival analysis is to estimate, interpret and compare survivor and/or hazard functions between different cohorts from survival data. The Cox-PH model^3^ is the most widely used survival analysis technique with the additional advantage of incorporating covariates.

The Cox-PH model is described as follows:

$$\ln\left\{\frac{h\left(t\right)}{h_{0}\left(t\right)}\right\}=b_{1}P_{1}+b_{2}P_{2}+\ldots +b_{n}P_{n},$$

where *h*(*t*) is the expected hazard at time *t*, *h*_0_(*t*) is the baseline hazard, and *b_1_*, …, *b_n_* are the regression coefficients for each predictor (or confounding factors) *P_1_*, …, *P_n_*, respectively.

To examine the potential temporal correlation for the disease pair *X* → *Y*, the event of interest in our study is the diagnosis of disease *Y*. In addition, both continuous and categorical confounding factors may be easily incorporated in Cox-PH model. For example, one of the predictors may be a categorical variable indicating whether the subject is exposed to disease *X* or not. Other confounding factors may include age, Body Mass Index (BMI), among others. The results of Cox-PH regression may examine whether the diagnosis of disease *Y* occurs sooner in the exposed group relative to the comparison group. The estimated coefficient, and its corresponding *P*-values will be extracted from the model. Benjamini-Hochberg multiple test correction method will be applied to *P*-values for all disease pairs. Adjusted *P*-values less or equal to 0.05 are deemed statistically significant.

### Quality controls for evaluating Cox-PH regression results

When applying any statistical models, it is critical to verify whether the data meet the assumptions of the underlying models. Two standard procedures have been implemented into the package for testing whether the major assumption of the constant hazard ratio for Cox-PH models is satisfied: (i) The *Cox.zph* method performs a statistical test with *P*-value < 0.05 indicating that the hazards are not proportional, (ii) The Cox-Snell residual plots used for visually assessing the fit of the Cox-PH models: if the model fits the data, the residual plots should follow the diagonal line.

**Figure 2.**
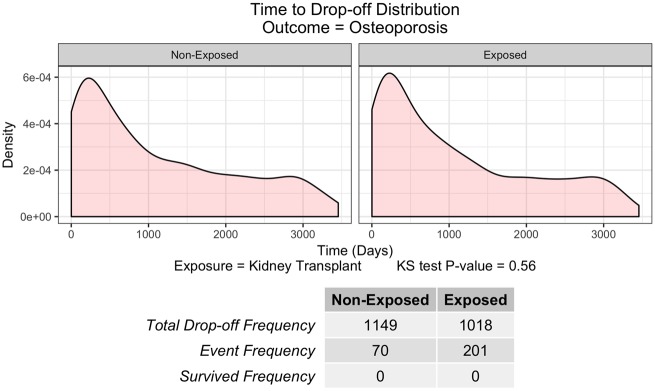
Time to Drop-off Distribution for disease pair: Kidney transplant to Osteoporosis. *X* axis denotes the time in days when the drop-off happens, while *Y* axis denotes the density function of drop-off distribution. Bottom frequency table shows the total drop-off frequency, event frequency, and survival frequency during the study period (10 years).

In addition to assessing the fit of the Cox-PH models, we have also recognized that not every randomly selected subject in our study is able to be followed for a predefined period, which can be considered a drop-out in survival analysis. Therefore, we also provide a novel check to apply the Kolmogorov–Smirnov test (KS test) to examine whether the number and timing of dropouts is similar between the exposed and comparison groups (Figure 2). If the data fails to pass the KS test (*P*-value <0.05), users should interpret the survival analysis results with caution.

### An independent machine-learning approach to survival analysis

Although Cox-PH regression is the most widely used method for survival analysis, it still relies on a restrictive assumption of proportional hazards. In addition, Cox-PH models rely on linear combinations of predictors, making it difficult to deal with non-linear effects and interactions among multiple predictors. To handle these difficulties, we have applied an independent machine-learning method of RF survival analysis.^4^ In brief, the machine-learning method builds survival trees based on random bootstrap samples. Each internal tree node is split by randomly selecting a subset of predictor variables which maximize survival differences between the two daughter nodes based on the log-rank splitting rule. The conditional cumulative hazard function is then estimated using the standard Nelson-Aalen estimator from terminal nodes of each tree and averaged over all trees.

We integrated the R package randomForestSRC^4^ in our method for RF survival analysis. However, the package for the RF survival analysis only produces predicted survival probability curve for each individual subject. Consequently, the standard log-rank test comparing two cohort-based survival curves (eg, exposed vs. comparison) cannot be applied directly. We overcame this statistical challenge as follows: It was shown that the average survival time is equal to the integral of the survival function.^5^ According to the central limit theorem, the average survival times from each individual subject follow a normal distribution. Therefore, we can perform a standard two-sample hypothesis test to compare the average survival time in the exposed cohort to the comparison cohort. Even though a parametric *t-*test could be applicable here, we chose to apply the non-parametric Wilcoxon rank-sum test to be conservative. Benjamini–Hochberg multiple test correction method will be applied to both *t*-test and Wilcoxon rank-sum test *P*-values for all disease pairs. Adjusted *P*-values less or equal to 0.05 are deemed statistically significant.

### Exploration of disease correlation networks

**Figure 3.**
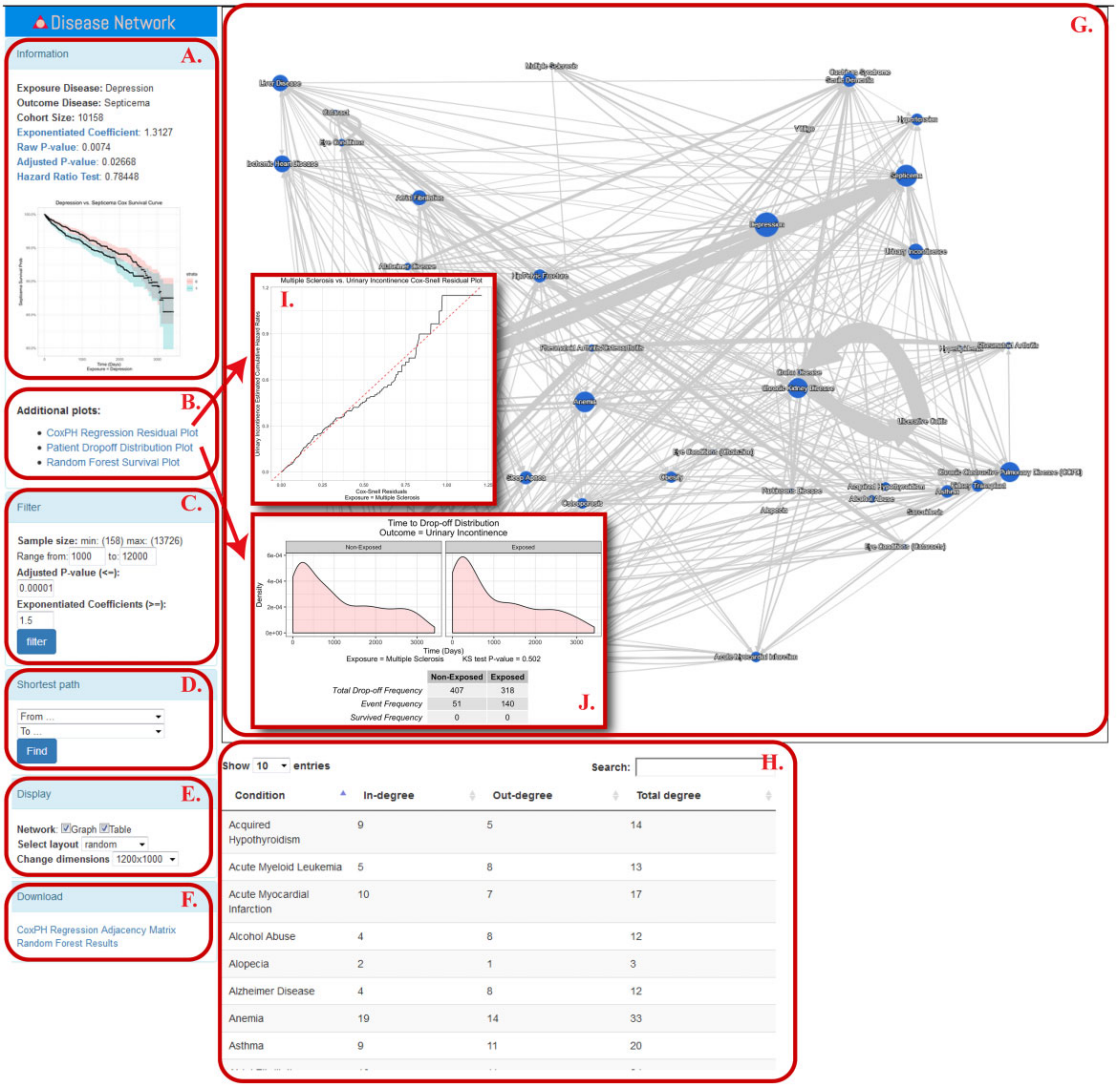
Screenshot of the interactive network display. Panel A—“Information” shows the exposure disease, outcome disease, coefficient, raw and adjusted *P*-values, hazard ratio test *P*-value, and a mini-sized survival plot. Panel B lists out additional evaluation plots which can be found through the links below. Panel C—“Filter” panel provides the users with options to filter edges based on sample size, adjusted *P*-values and coefficients. Panel D—“Shortest Path” finds the shortest path between any two given diseases. Panel E—“Display” provides several options to display the network in Panel G. Panel F provides download links for both Cox-PH regression and RF survival analysis results in CSV format. Panel G displays the interactive disease network based on Cox-PH regression result. Panel H shows in-degree, out-degree and total degree information for all disease nodes shown in the network. Users can search for a disease of interest through the search box. Panel I provides an example of a Cox-Snell residual plot, while Panel J provides an example of a drop-off distribution plot. More instructions on how to use the network visualization tool are available at https://github.com/qunfengdong/DCN.

The output from the previous steps is displayed as a directional DCN in which each disease is displayed as a node and connections with arrows are placed between nodes indicating directional statistically significant correlations (Figure 3, Panel G). The network display is implemented using the Cytoscape JS package.^6^ Besides displaying in-degree, out-degree and overall-degree of a node (Figure 3, Panel H), users may also filter the network based on multiple test corrected *P*-value cutoffs and explore trajectories in the network between any two nodes based on shortest-path in graph theory (Figure 3, Panel C-E). When clicking on each connection, additional information appears at the left panel (Figure 3, Panel A-B), such as *P*-value of the hazard ratio test, survival plots, Cox-PH regression residual plots, patient drop-off distribution plots, and RF survival plots.

## RESULTS AND DISCUSSION

**Figure 4.**
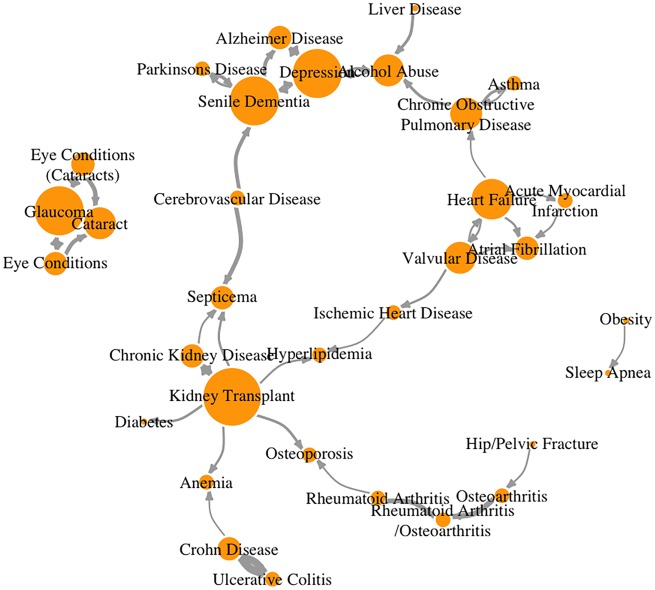
Network display of the selected statistically significant disease pairs. Each disease is a node (orange). Node size is proportional to its in-degree. Temporal relationships between disease pairs are edges (gray) with arrow heads denoting direction. The thickness of the edges are proportional to their coefficients.

We have applied our method to a longitudinal de-identified EMR dataset at Loyola University Chicago Medical Center with 654 084 distinct initial disease encounters relating to 51 conditions in 175 539 patients. In total, 321 pairs of disease states were found to be temporally associated within 10 years using Cox-PH regression after multiple test correction. The independent RF survival analysis identified 449 pairs of disease states to be statistically temporally associated according to both the adjusted Wilcoxon ranked-sum test and two-sample *t*-test *P*-values. There were 298 overlapping disease pairs identified by both methods, which were selected for display in the interactive network for this study. Although we elected to focus on overlapping pairs due to the higher confidence imposed by concordance between two independent methods, users may choose to utilize each method alone (those results are also part of the outputs produced by DCN). The results for this study are available for download and review at http://cbi.lumc.edu/disease/, in which nodes indicate diseases and directional arrows indicate temporal correlations between diseases. Over 90% of the temporal correlations identified by our software are supported by literature review. In other words, our software was able to identify many known temporal correlations among diseases from EMR data without any prior knowledge. Table 1 shows a few examples of temporal correlations identified by our software, all supported by the corresponding literature. A network plot is presented in Figure 4 to provide a graphical representation.

**Table 1.** Example disease pairs of statistically significant temporal relationship detected by DCN

From-disease	To-disease	Adjusted hazard ratio	Cohort size	HR test *P*-value	Cox-PH regression *P*-value (Bonferroni adjusted)	Literature support
Acute myocardial infarction	Atrial fibrillation	2.273	2002	0.198	1E–05	^8^
Alcohol abuse	Depression	2.398	2912	0.009	3E–24	^9^
Alzheimer disease	Depression	2.030	2058	0.708	2E–07	^10^
Asthma	Chronic obstructive pulmonary disease	2.255	9548	0.053	2E–24	^11^
Cataract	Glaucoma	2.495	9482	0.013	2E–31	^12^
Cerebrovascular disease	Septicemia	6.164	1098	0.821	9E–08	No obvious literature support
Cerebrovascular disease	Senile dementia	4.879	1098	0.569	2E–08	^13^
Chronic kidney disease	Kidney transplant	9.094	9622	0.735	9E–39	Well-established relationship
Chronic kidney disease	Septicemia	2.281	9622	0.916	5E–20	^14^
Chronic obstructive pulmonary disease	Alcohol abuse	3.490	10322	0.474	7E–09	^15^
Chronic obstructive pulmonary disease	Asthma	3.364	10 322	0.016	4E–99	^16^
Crohn disease	Ulcerative colitis	22.203	1492	0.538	5E–09	^17^
Crohn disease	Anemia	2.037	1492	0.309	7E–11	^18^
Depression	Alcohol abuse	2.853	10 158	0.448	6E–08	^19^
Depression	Alzheimer disease	2.799	10 158	0.636	2E–08	^10^
Depression	Senile dementia	2.065	10 158	0.480	9E–10	^20^
Eye conditions (cataracts)	Glaucoma	2.961	12 850	0.000	1E–70	^13^
Glaucoma	Cataract	2.269	7092	0.544	6E–40	^21^
Heart failure	Acute myocardial infarction	2.243	11 764	0.849	1E–08	^22^
Heart failure	Chronic obstructive pulmonary disease	2.032	11 764	0.966	7E–35	^23^
Ischemic heart disease	Hyperlipidemia	2.152	12 618	0.000	9E–146	^24^
Kidney transplant	Osteoporosis	3.202	2438	0.764	2E–14	^7^
Kidney transplant	Diabetes	3.009	2438	0.000	7E–39	^25^
Kidney transplant	Septicemia	2.758	2438	0.506	1E–08	^26^
Kidney transplant	Anemia	2.741	2438	0.000	1E–46	^27^
Kidney transplant	Hyperlipidemia	2.426	2438	0.007	4E–46	^28^
Obesity	Sleep Apnea	2.072	11 202	0.002	5E–36	^29^
Senile dementia	Parkinson’s disease	2.949	5088	0.662	6E–06	^30^
Senile dementia	Depression	2.194	5088	0.077	2E–22	^31^
Ulcerative colitis	Crohn disease	29.319	1622	0.975	2E–13	^17^
Valvular disease	Atrial fibrillation	2.455	12 690	0.000	3E–70	^32^

*Note*: From left to right, it lists the exposure disease (From-Disease), outcome disease (To-Disease), the confounding factor adjusted hazard ratio estimated from Cox-PH regression, cohort size, hazard ratio test for proportional hazards assumption for Cox regression, Bonferroni adjusted Cox-PH regression *P*-value, and literature support or evaluation of the relationship for this disease pair.

As an example, our results showed that patients who had kidney transplant earlier in life have a higher risk of developing osteoporosis within 10 years. The estimated hazard ratio of having osteoporosis after kidney transplant is 3.2, which indicates a significant higher risk of getting osteoporosis in patients who had a kidney transplant than those who had other conditions when holding confounding factors such as age, gender, etc. constant. Under closer examination of the assumption for the Cox-PH regression, the hazard ratio test *P*-value is 0.764, indicating that the assumption of proportional hazards for Cox-PH regression was not violated. Additionally, KS test was performed to inspect whether the number and timing of drop-out in the two populations had similar distribution. The KS test showed a *P*-value of 0.56 (Figure 2), which indicates that the drop-off distributions in the exposed and unexposed groups are not different from each other. With the confidence supported by the Cox-PH assumption and drop-off distribution check, we hypothesize that there is a strong temporal correlation between kidney transplant and osteoporosis. The independent RF survival analysis also supports the hypothesis with both two-sample *t*-test and Wilcoxon rank-sum test adjusted *P*-value less than 0.0001. The RF method estimated the mean survival time to be 3009 days in the exposed group and 3279 days in the nonexposed group, meaning that on average, people who had kidney transplants develop osteoporosis about 9 months (3279–3009 days) sooner than those who did not. The literature^7^^,^^33^ also confirms that about 10% to 56% of the patients with kidney transplants have accelerated bone loss.

Large-scale EMR datasets present exciting opportunities for making novel correlations between chronic disease states, but do hold certain limitations, particularly in historic data sets. For Cox-PH regression, we have implemented several quality controls in our package such as KS test and Cox-Snell residual plot to help aid the users in determining whether a detected significant disease correlation is reliable. Investigators must maintain caution when attempting to draw definite conclusions due to the intrinsic limitations of EMRs. We emphasize that DCN is intended for the formulation of innovative research hypotheses rather than firm conclusions. Once a hypothesis is generated from DCN results, a future study may be designed to further characterize the relationship, assess its validity, and take into consideration comorbidities, covariates, and other confounding factors.

Investigators who are comfortable with command-line tools are encouraged to use our software to perform data mining on their own large-scale EMR data in various ways (eg, by exploring different parameter values other than default to uncover novel temporal correlations between disease states). Those uncovered correlations should be utilized to create novel research hypotheses, which may be further investigated in prospective studies.

In conclusion, we have produced an easy-to-use software by integrating Cox-PH regression and RF survival analysis to identify temporal correlations between disease states. We also provide quality control tools to help evaluate the reliability of these results. Over 90% of the detected statistically significant disease pairs with temporal correlations are supported by published literature.

## FUNDING STATEMENT

This research received no specific grant from any funding agency in the public, commercial or not-for-profit sectors.

## COMPETING INTERESTS STATEMENT

The authors have no competing interests to declare.

## CONTRIBUTORSHIP STATEMENT

Huaiying Lin: algorithm development, software implementation and testing, data analysis, manuscript drafting and revision; Ruichen Rong: algorithm development, software implementation and testing; Xiang Gao: algorithm development and data analysis; Kashi Revanna: software implementation; Michael Zhao: software implementation and testing; Petar Bajic: manuscript drafting and revision; David Jin: software implementation and testing; Chengjun Hu: data analysis; Qunfeng Dong: project conception, algorithm development, data analysis, manuscript drafting and revision.
